# Aberrant LncRNA Expression in Leukemia

**DOI:** 10.7150/jca.42093

**Published:** 2020-04-27

**Authors:** Jie Gao, Fujue Wang, Pengqiang Wu, Yingying Chen, Yongqian Jia

**Affiliations:** Department of Hematology, West China Hospital, Sichuan University, Chengdu 610041, Sichuan, China

**Keywords:** long non-coding RNAs (lncRNAs), leukemia, transcription regulation, expression profiling, therapeutic targets

## Abstract

Leukemia is a common malignant cancer of the hematopoietic system, whose pathogenesis has not been fully elucidated. Long noncoding RNAs (lncRNAs) are transcripts longer than 200 nucleotides without protein-coding function. Recent studies report their role in cellular processes such as the regulation of gene expression, as well as in the carcinogenesis, occurrence, development, and prognosis of various tumors. Evidence indicating relationships between a variety of lncRNAs and leukemia pathophysiology has increased dramatically in the previous decade, with specific lncRNAs expected to serve as diagnostic biomarkers, novel therapeutic targets, and predictors of clinical outcomes. Furthermore, these lncRNAs might offer insight into disease pathogenesis and novel treatment options. This review summarizes progress in studies on the role(s) of lncRNAs in leukemia.

## Introduction

Leukemia is a malignant clonal disease of hematopoietic stem and progenitor cells, in which abnormally cloned leukemia cells accumulate in the bone marrow and other non-hematopoietic tissues owing to uncontrolled cell proliferation, blocked differentiation, and apoptosis obstruction, thus inhibiting normal hematopoiesis and immune function. Rapid advances in cell and molecular biology have enabled the discovery of dysregulated molecules associated with leukemia, suggesting that the disease might be related to the heterogeneity of cellular and molecular genetics 1-4. Chromosomal abnormalities, including the appearance of hyperdiploid and hypodiploid characteristics, amplification, translocation, changes in DNA copy number, as well as insertion, deletion, and point mutations, are commonly observed in leukemia 5. Moreover, modifications of transcription factors, tumor-suppressor genes, oncogenic mutations, and epigenetic changes have been reported 6; however, the specific pathogenesis of leukemia remains poorly understood.

**Figure 1:**
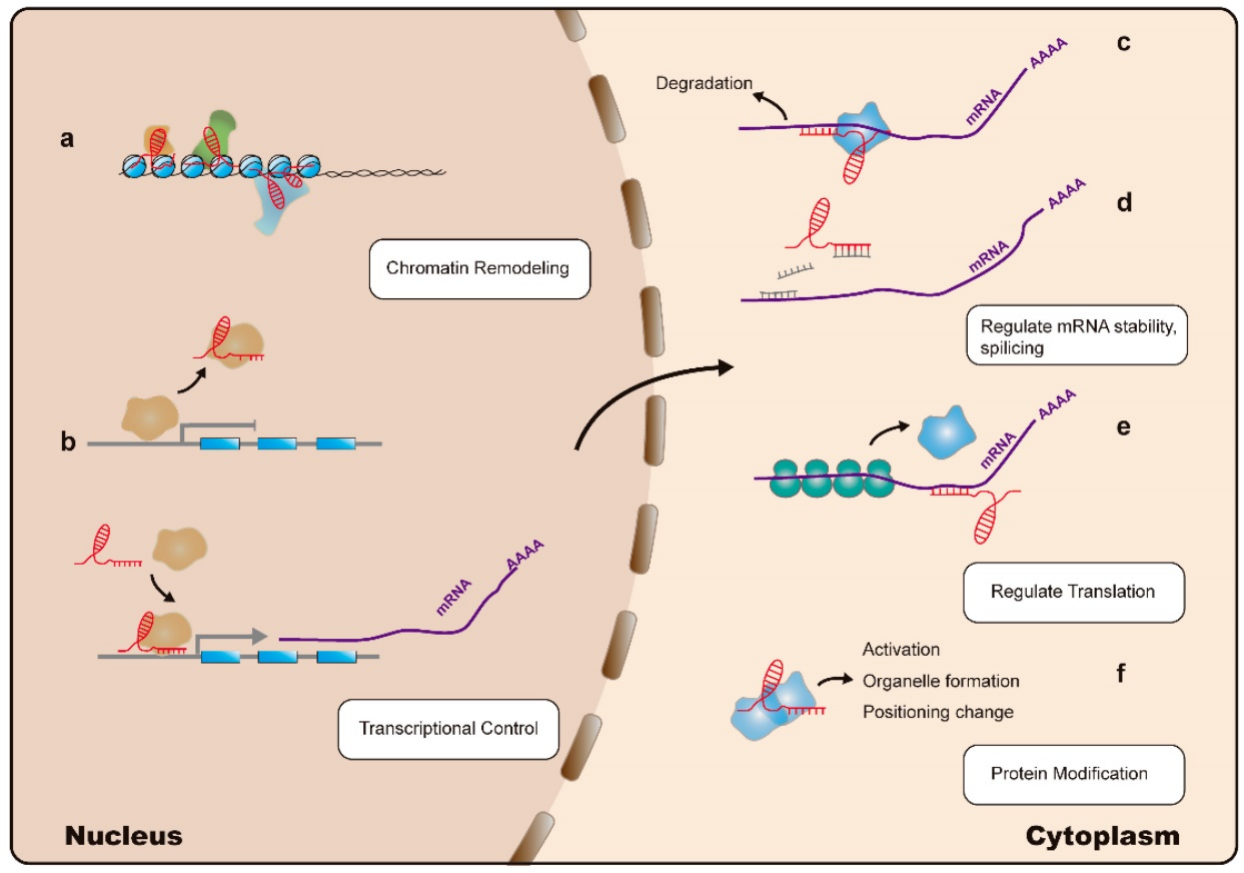
Mechanisms of LncRNA action: lncRNAs (indicated in red) regulate gene expression at multiple levels: (a). LncRNAs can interact with the nuclear chromatin remodeling complex to achieve epigenetic regulation of target loci. (b). LncRNAs can regulate transcription by acting as a decoy or guide for transcription factors (indicated in yellow), thereby inhibiting or promoting their binding to target promoter sequences, respectively. (c). LncRNAs can interact with Staufen homolog proteins, thereby regulating mRNA stability. (d). LncRNAs can modulate mRNA levels by competing for microRNA (indicated in grey) binding. (e). Translation of mRNA can be modulated by lncRNAs. (f). LncRNAs can directly alter protein (indicated in blue) functions.

Less than 2% of the human genome encodes proteins, with the remaining 98% considered as genetic byproducts. However, studies demonstrate that areas of the genome are transcribed as noncoding RNAs 7, 8; these include various types of small-noncoding RNAs such as microRNAs (miRNAs), small-interfering (si)RNAs, small-nucleolar RNAs, and piwi-associated RNAs. Some dysregulated noncoding RNAs, particularly miRNAs, are well-known gene silencers 9. Among noncoding RNAs, long noncoding (lnc)RNAs are a class of transcripts with a length >200 nucleotides and no protein-coding function. It was thought that lncRNAs lack biological functions; however, recent application of high-throughput sequencing and the rapid development of biological techniques have resulted in lncRNAs becoming a subject of tremendous research interest. Increasing evidence indicates that lncRNAs can regulate gene expression at multiple levels, including the epigenetic, transcriptional, and post-transcriptional stages. Additionally, lncRNAs are considered to be involved in the induction of chromatin remodeling and nucleosome modification, transcriptional activation and inhibition, regulation of variable splicing modes and protein activity, generation of endogenous siRNAs, and changing the protein localization (Figure 1) 10-12. Moreover, lncRNAs are involved in the pathogenesis of cancer-related diseases, and therefore represent potential biomarkers and therapeutic targets 11. Additionally, studies on the relationship between lncRNAs and hematological malignancies, such as leukemia, multiple myeloma, and lymphoma, are increasing. A variety of lncRNAs with potential as oncogenes or tumor-suppressors have been identified as significantly associated with the development and progression of these diseases 13-15.

This review summarizes the current knowledge regarding lncRNA involvement in leukemia. The data indicate that several lncRNAs might have clinically promising applications in the diagnosis, prognosis, and treatment of leukemia.

## Overview of LncRNAs

LncRNAs exhibit high functional heterogeneity, are generally located in the cytoplasm or nucleus, and possess diverse biological functions and complex regulatory mechanisms. According to the relative positional relationship between protein-coding genes and lncRNAs, lncRNAs can be roughly classified into five types: intron lncRNAs, antisense lncRNAs, intergenic lncRNAs, UTR (untranslated region)-associated lncRNAs, and promoter-associated lncRNAs 16. Functionally speaking, lncRNAs can regulate gene expression at the chromatin-modification, gene-transcription, and post-transcriptional levels. Furthermore, mechanistically, lncRNA activity can be classified into four modes: signal, decoy, guide, and scaffold 17. In the signal mode of action, lncRNAs can be associated with the genome-imprinting process: for example, when two X chromosomes are present, one is in a suppressed state, with this phenotype capable of being stably transmitted to a subsequent generation, where the X-inactive-specific transcript (Xist) plays an important regulatory role 18, 19. The mode in which lncRNA functions as a decoy can be described as the binding of lncRNA to a protein with transcription-regulatory functions, such as transcription factors or nuclear receptors in the nucleus, in order to regulate transcriptional inhibition of target genes downstream. For example, when DNA damage occurs, p53 binds to the cyclin-dependent kinase inhibitor (CDKN)1 and activates expression of the lncRNA PANDAR, which blocks the expression of pro-apoptotic genes by binding to the nuclear transcription factor Y subunit α (NF-YA), thus prolonging cell survival 20. As a guide, lncRNA binds to a protein molecule (usually a transcription factor) and promotes its localization to a specific DNA sequence to regulate downstream signaling pathways and gene expression. An example of this is the lncRNA Xist acting as a guide to target gene-silencing activity in an allele-specific manner. As a scaffold, lncRNA binds to multiple effector molecules simultaneously to provide a platform for interaction. For example, the 3′ domain of the lncRNA HOX antisense intergenic RNA (HOTAIR) binds the histone demethylase complex lysine-specific demethylase (LSD)1/CoRE1-silencing transcription factor (REST)/REST, whereas the 5′ domain of HOTAIR binds Polycomb repressive complex 2 (PRC2). The resulting interaction promotes assembly of selected histone-modification enzymes by providing a binding surface, thereby resulting in the chromosome being in a closed state; this results in gene silencing 21, 22. In this review, we focused on the functions and mechanisms of lncRNAs involved in leukemia pathogenesis (Table 1).

## Aberrant LncRNA Expression in Leukemia

**Figure 2:**
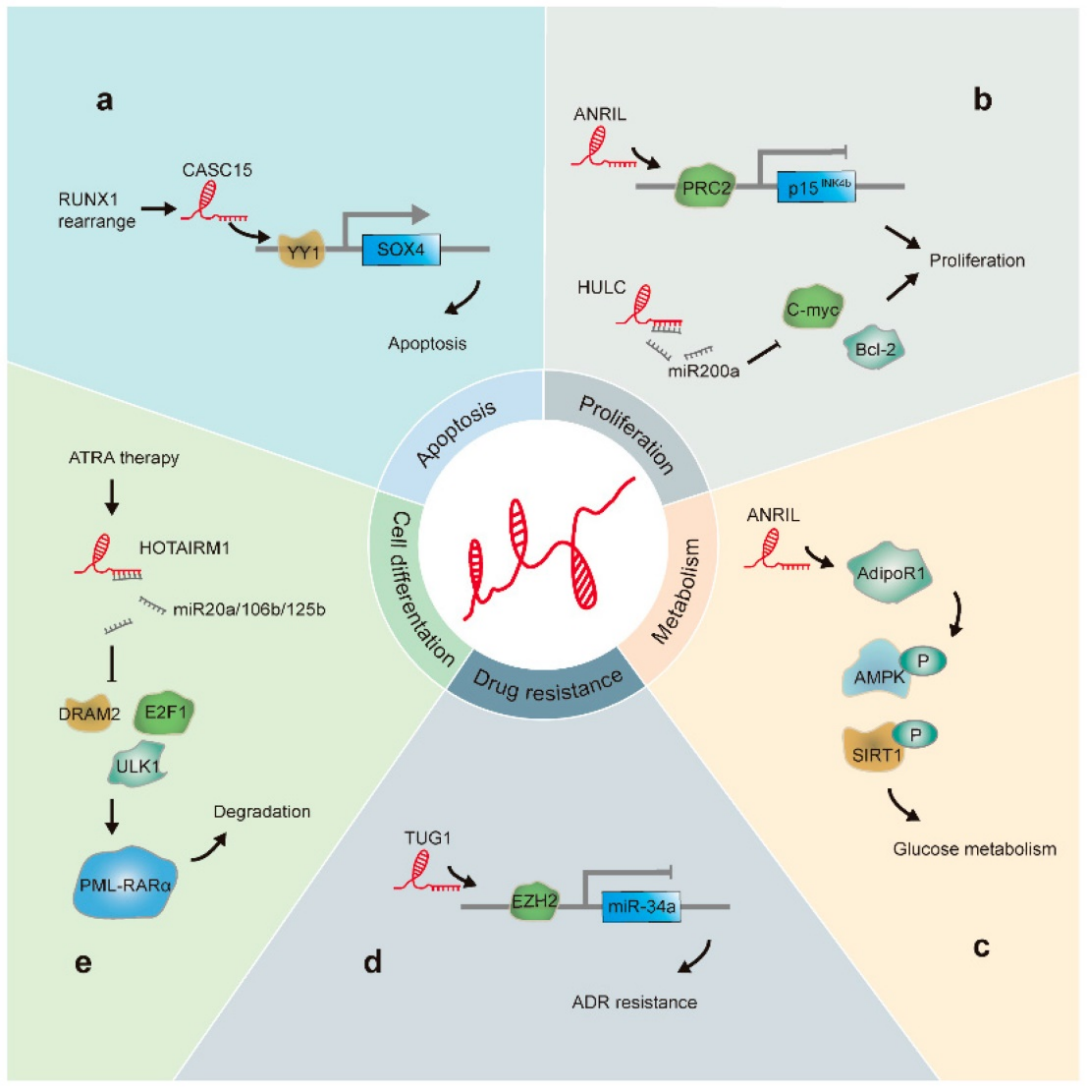
LncRNAs involved in leukemia progression: (a). CASC15 is upregulated in RUNX1-rearranged AML. Additionally, CASC15 enhances Yin and Yang-1(YY1)-mediated regulation of the SOX4 promoter, thus increasing apoptosis. (b). HULC acts as a sponge for miR-200a and modulates c-Myc and Bcl-2 levels, promoting CML cell proliferation. ANRIL recruits PRC2 to the p15^INK4b^ locus and silences the p15^INK4b^ tumor suppressor gene, resulting in cell proliferation. (c) ANRIL regulates the expression of the adiponectin receptor (AdipoR1), a key regulator of glucose metabolism; this results in the regulation of AMPK and SIRT1 phosphorylation levels. (d). TUG1 epigenetically suppresses miR-34a expression by increasing EZH2 recruitment and H3K27me3 levels at the miR-34a promoter in AML cells, thus contributing to ADR resistance. (e). HOTAIRM1 acts as a sponge for miRNA20a/106b/125b, regulates the expression of autophagy-associated genes, and enhances PML-RARα degradation.

The positive or negative role of lncRNAs in leukemia progression is determined by their activity in terms of their specific roles in differentiation, energy metabolism, malignant proliferation, apoptosis, and drug resistance of leukemia cells (Figure 2). Here, we focused on lncRNAs that have been well studied in association with leukemia and describe the progress in this field and mechanisms elucidated.

### Maternal Expression Gene 3 (MEG3)

MEG3, a putative tumor-suppressor gene located on chromosome 14q32, suppresses the proliferation of various tumor cells by directly regulating retinoblastoma protein phosphorylation and indirectly activating the p16^INK4a^ pathway^23^, ^101^. Benetatos et al. observed that, in a sample of 42 acute myelogenous leukemia (AML) patients, 47.6% of patients displayed hypermethylation of MEG3, with MEG3 methylation associated with significantly reduced overall survival (OS); these findings suggest that this methylation status represents a useful biomarker for leukemia 24. Previous studies have found that MEG3 plays a regulatory role in carcinogenesis and metastasis in chronic myelogenous leukemia (CML) by interacting with miRNA-21 76. Li et al. found that patients in accelerated and blast phases showed lower expression of miR-147 and MEG3. Furthermore, it was shown that MEG3 was capable of binding to several members of the Janus kinase (JAK)-signal transducer and activators of the transcription (STAT) pathway, resulting in reduced signaling. This activity regulated leukemia progression, suggesting a role for MEG3 and its target miR-147 as new therapeutic targets 77. Furthermore, Zhou et al. showed that MEG3 might be involved in imatinib resistance by regulating miR-21, thereby affecting cell proliferation, multidrug-resistance transporter expression, and cell apoptosis 78.

### Nuclear Paraspeckle Assembly Transcript 1 (NEAT1)

NEAT1 is located on chromosome 11 and encodes transcripts that localize specifically to nuclear paraspeckles 102. Studies show that NEAT1 is overexpressed in several types of solid tumors including childhood ALL samples^103^; in addition, NEAT1 is associated with aberrant expression of miR-335-3p, resulting in indirect modulation of the expression of multidrug-resistant genes, including ATP-binding cassette subfamily A member 3 62. However, in certain types of cancers, such as AML, NEAT1 might act as a tumor suppressor. Zhao et al. showed that the expression of NEAT1 and structural maintenance of chromosome 1α (SMC1A) were decreased in primary AML patients and THP-1 cells. Additionally, they found elevated levels of miR-23a-3p, which indicate that NEAT1 binds miR-23a-3p to regulate SMC1A expression, thereby inhibiting leukemia cell proliferation and enhancing apoptosis 54. In de novo acute promyelocytic leukemia (APL), NEAT1 levels are significantly reduced: Zeng et al. reported that the promyelocytic leukemia-retinoic acid receptor α (PML-RARα) fusion protein inhibits NEAT1 expression, while the latter is significantly upregulated in APL cells treated with all-trans retinoic acid (ATRA) 104. This group subsequently found downregulated NEAT1 expression in primary CML cells, which was restored by inhibition of BCR-ABL kinase activity 84. Moreover, their results indicated that NEAT1 was transcriptionally regulated by c-Myc via binding to the NEAT1 promoter, and that splicing factor and proline- and glutamine-rich protein were required for NEAT1-mediated K562 cell apoptosis 84. These results suggest that targeting NEAT1 represents a new treatment strategy for leukemia, and contribute to a more comprehensive understanding of the pathogenesis of this cancer.

### HOX Antisense Intergenic RNA Myeloid 1 (HOTAIRM1)

HOTAIRM1 is a lncRNA located in the HOXA gene cluster; this lncRNA is generated by RNA polymerase II antisense transcription and expressed in the myeloid lineage. Retinoic acid induces HOTAIRM1 expression and thereby regulates the expression of genes involved in myeloid differentiation. Studies show that HOTAIRM1 levels are significantly elevated during ATRA-induced NB4 cell lineage differentiation; in contrast, HOTAIRMI knockdown inhibits ATRA-induced granulocyte differentiation and releases cell cycle arrest in the G1/S phase, revealing that HOTAIRM1 can regulate the maturation of myeloid cells by affecting integrin gene expression 105. Additionally, Chen et al. revealed that HOTAIRM1 acts as a miRNA sponge for miR-20a/106b and miR-125b. Further, downregulation of HOTAIRM1 levels inhibits ATRA-induced PML-RARα degradation via miRNA-mediated pathways to suppress the expression of autophagy-related genes and granulocyte differentiation of APL cells 106. These results suggest that HOTAIRM1 plays an essential role in myeloid differentiation in leukemia. However, another study showed that in 241 AML patient specimens, elevated HOTAIRM1 levels were associated with shorter leukemia-free survival, shorter OS, and higher cumulative incidence of relapse 106.

### Leukemia-induced Noncoding Activator RNA-1 (LUNAR1)

LUNAR1 is a NOTCH-regulated oncogenic lncRNA, located on chromosome 15q26.3, and specifically expressed in T cell acute lymphoblastic leukemia (T-ALL), thereby playing a crucial role in its progression. The gene encoding LUNAR1 is located in close proximity to the insulin-like growth factor 1 receptor (IGF1R) gene. Upon activation, LUNAR1 recruits the mediator complex on the IGF1R promoter to regulate its transcription, thereby promoting T-ALL cell proliferation 67. In vivo experiments involving the transplantation of tumor cells into mice revealed that tumor proliferation was blocked only in mice in which LUNAR1 was inactivated. This suggests that LUNAR1 inhibitors can potentially be used for targeted therapy.

### Antisense Noncoding RNA in the INK4 Locus (ANRIL)

ANRIL, which is located on chromosome 9p21, exerts an oncogenic function through modulation of p15^INK4b^ and p16^INK4a^ expression^107^. In the development of B-cell precursor (BP)-ALL and AML, ANRIL is overexpressed, thereby aggravating inhibition of the p15^INK4b^ locus. Iacobucci et al. compared ALL blood samples with nonmalignant controls and showed an apparent correlation between ANRIL and BCR-ABL-associated ALL nucleotide polymorphisms. They speculated that this association reflects the ability of certain ANRIL polymorphisms to contribute to their own transcriptional alteration and increased susceptibility to ALL^69^. In AML, ANRIL is upregulated in patients and downregulated after complete remission (CR) 106. Additionally, this study showed that ANRIL regulated the expression of the adiponectin receptor and its downstream factors adenosine monophosphate-activated protein kinase (AMPK)/sirtuin 1 (SIRT1), thereby modulating disease progression by regulating glucose metabolism 108; these findings suggest that ANRIL represents both a potential candidate for AML diagnosis and a therapeutic target. Furthermore, Song et al. showed that ANRIL could promote the proliferation of T-ALL cells by targeting enhancer of zeste homolog 2 (EZH2) and activating the nuclear factor kappaB (NF-κB) pathway, indicating that aberrant ANRIL expression was involved in T-ALL leukemogenesis 70.

### Deleted in Leukemia (DLEU)1/2

Over 50% of patients with chronic B-cell lymphocytic leukemia have a 13q14.3 deficiency. This critical deleted region comprises two adjacent subregions: DLEU1 and DLEU2 109. DLEU2 negatively regulates cyclins D1 and E1 via miR-15a and miR-16-1, which play important roles in chronic lymphocytic leukemia (CLL) pathogenesis by regulating B-cell lymphoma 2 expression 92, 110. Additionally, Garding et al. found that DLEU1 and DLEU2 were significantly demethylated at their respective 5′ end in almost all CLL patients, resulting in attenuated transcription of a series of adjacent sequences encoding tumor-suppressor genes 93. Moreover, DLEU1 is reportedly poorly expressed in other solid tumors and negatively correlated with prognosis 111. Therefore, its clinical application in leukemia treatment requires further investigation.

### β Globin Locus Transcript 3 (BGL3)

BGL3 is located on chromosome 11p15.4 and negatively regulated by c-Myc-dependent DNA methylation. BGL3 is a host of miR-17, miR-20a, miR-20b, miR-93, miR-106a, and miR-106b, and acts as a competing endogenous (ce)RNA to alter the expression of the tumor suppressor PTEN. Additionally, BGL3 overexpression significantly reduces the survival of K562 cells and promotes imatinib-induced apoptosis 90. These results indicate that BCR-ABL-mediated cell transformation requires silencing of the tumor suppressor BGL3, thereby offering a potential strategy for treatment of BCR-ABL-positive leukemia.

### HOX Transcript Antisense RNA (HOTAIR)

HOTAIR is located on chromosome 12 and is transcribed from the antisense strand of the homeobox C gene locus. HOTAIR plays a repressive role by interacting with and guiding various chromatin-modifying complexes, including LSD1 and PRC2, to target-gene promoter regions, resulting in gene silencing 112. In hematological malignancies, HOTAIR regulates self-renewal of leukemia stem cells (LSCs) to promote uncontrolled self-renewal and proliferation 33. Previous studies report HOTAIR overexpression in AML patients, and show that these elevated levels are associated with higher peripheral leukocyte and bone marrow blast counts and lower platelet and hemoglobin counts, as well as poor clinical prognosis 29-31. Furthermore, HOTAIR acts as a ceRNA by binding to miR-193a, which targets c-KIT, thus modulating c-KIT expression 32. In CML, HOTAIR is upregulated in patients with elevated levels of multidrug-resistance protein 1. In K562 imatinib-resistant cells, HOTAIR knockdown leads to higher sensitivity to imatinib via the inactivation of phosphoinositide 3-kinase (PI3K)/AKT signaling 75. These results suggest HOTAIR is involved in the development of imatinib resistance.

### Urothelial Carcinoma-associated 1 (UCA1)

**Figure 3:**
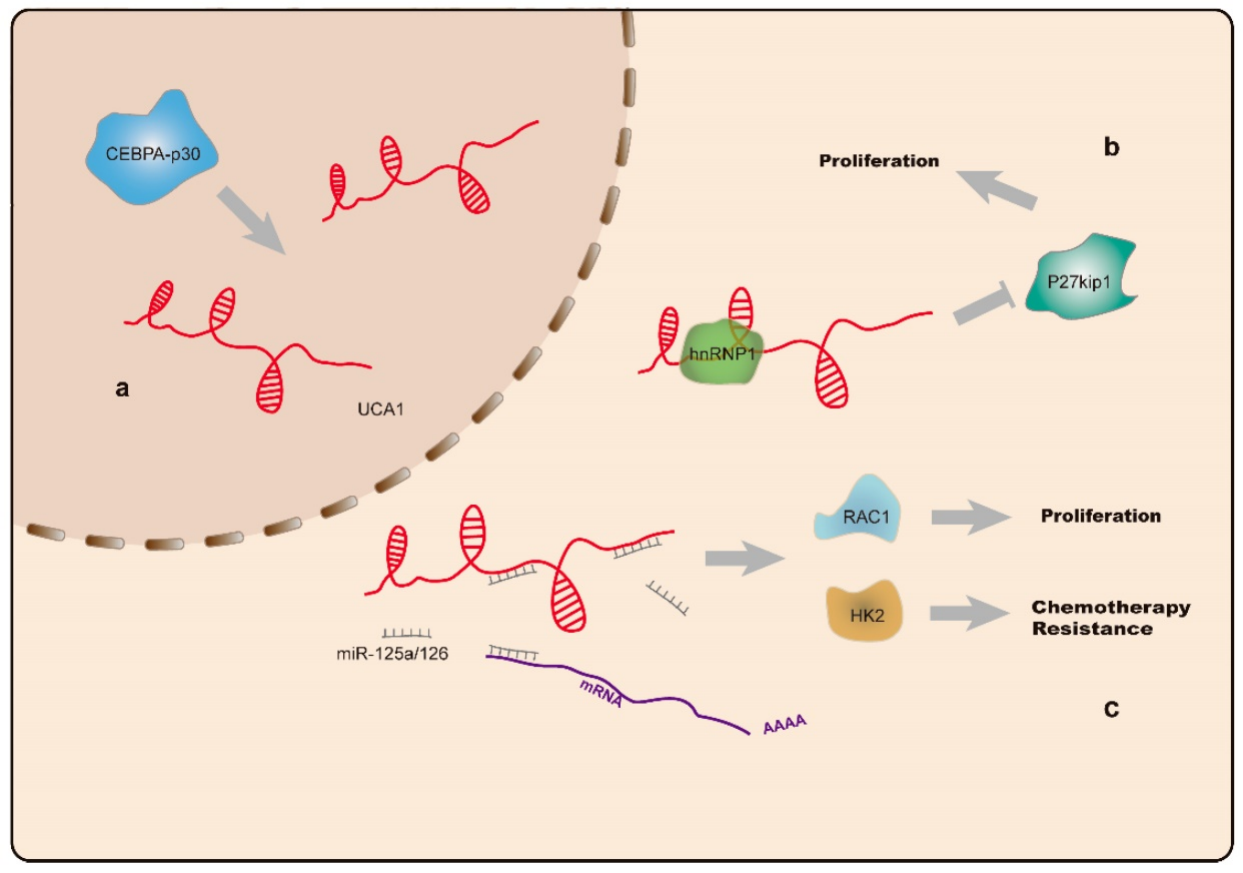
a. CEBPA-p30 protein promotes UCA1 (indicated in red) expression in AML cells with CEBPA mutations. b. UCA1 sustains proliferation of AML cells by repressing the expression of the cell cycle regulator p27^kip1^. c. The UCA1 transcript functions as a sponge for miR-125a and miR-126, thus modulating RAC1 and HK2 expression, and promotes AML cell proliferation and resistance to chemotherapy.

UCA1 is located on chromosome 19p13 113 and highly expressed as a proto-oncogene in a variety of tumors 114-118. Additionally, UCA1 levels are elevated in AML and CML cell lines. The oncogenic effect of UCA1 is achieved by sponging miR-126, which precludes degradation of Ras-related C3 botulinum toxin substrate-1(RAC1) and activates JAK/STAT and PI3K/AKT signaling 26. Hughes et al. found elevated UCA1 levels in AML patients carrying CCAAT enhancer binding protein α (CEBPA) mutations, and that UCA1 sustained the proliferation of AML cells by inhibiting expression of the cell cycle regulator p27^kip1^ 27. Additionally, they found that abnormally expressed UCA1 acted as a ceRNA targeting miR-125a, which resulted in upregulated hexokinase-2 (HK2) expression, a key enzyme involved in glycolysis. Moreover, UCA1 is associated with resistance to chemotherapy, with elevation of UCA1 expression following doxorubicin (ADR)-based chemotherapy. UCA1 knockdown in ADR-resistant HL60 cells partially reversed AML chemoresistance via the miR-125a/HK2 axis(Figure 3) 28. Another study reported that UCA1 is capable of binding miR-16 to regulate MDR1 expression and promote imatinib resistance in CML cells 87. These findings support UCA1 as a potential diagnostic biomarker and therapeutic target for leukemia treatment and reversal of drug resistance.

### H19

H19 is an endogenous gene located on chromosome 11p15 119. H19 maintains hematopoietic stem cell (HSC) quiescence at the transcriptional and post-transcriptional levels by regulating IGF2-IGFR1 activity 120. Other studies have identified H19 as either an oncogene or tumor suppressor, depending on tumor type 121-125. Guo et al. reported that H19 expression is positively regulated by c-Myc and required for tumorigenesis, as H19 knockdown enhanced the sensitivity of CML cells to imatinib, inhibited BCR-ABL-induced tumor proliferation, and promoted apoptosis 79. A subsequent study indicated that hypomethylation of the H19 differentially methylated region/imprinting control region might mediate its overexpression in CML 80. Zhang et al. identified upregulated H19 levels in AML patients that were correlated with lower CR rates and shorter OS 25. These findings suggest that H19 plays different roles in different malignancies. Therefore, further research is needed to comprehensively elucidate the H19-specific mechanisms of action in leukemia.

## LncRNA Expression Profiles in Leukemia

Although the study of global lncRNA expression in leukemia remains limited, the expression patterns of various lncRNAs related to leukemia tumorigenesis and specific subtypes have been examined through expression profile analysis.

Lei et al. performed transcriptome analysis of lncRNA-expression profiles of AML patient samples and healthy controls, and identified differentially expressed lncRNAs, revealing that upregulated lncRNAs in AML were related to higher levels of binding to transcription factors such as STAT4, SP1 and ELK1, and lower levels of DNA methylation. Additionally, they found that LOC285758 stimulates the proliferation of AML cell lines by increasing levels of histone deacetylase 2, with elevated LOC285758 levels in patients associated with worse prognosis 126. By comparing in-depth sequencing data for various RNA-seq libraries and integrating RNA-seq data from 179 AML patients, Zhang et al. showed that lncRNAs are associated with specific AML subtypes 127. They observed that a subset of lncRNAs were abundantly expressed in patients with M3 subtypes, which are initiated following expression of the PML-RARα fusion gene. Schwarzer et al. revealed noncoding RNA stem cell characteristics as prognostic features shared by healthy HSCs and AML blasts cells, and identified lncRNA signatures specific for acute megakaryoblastic leukemia (AMKL), Down syndrome-associated AMKL, inv(16), t(8; 21), and mixed-lineage leukemia-rearranged samples 128. Ghavazi et al. performed a comprehensive analysis of the lncRNA transcriptome in ETS variant 6 (ETV6)/Runt-related transcription factor 1 (RUNX1)-positive BP-ALL, and found a specific lncRNA signature comprising 596 lncRNA transcripts 129 . Following data integration with RNA-seq results from other BP-ALL cell lines, they identified 16 unique lncRNA-expression profiles associated with the ETV6/RUNX1 fusion protein, including a potential carcinogenic lncRNA (DBH-AS1) 130. Another study revealed differential lncRNA expression between AML and ALL patients (1168 mRNAs and 2101 lncRNAs differed between leukemia subsets), with subsequent analysis of co-expression networks revealing single mRNAs potentially associated with more than one lncRNA, and vice versa. These results indicate that lncRNAs play important roles in regulating AML and ALL development 131.

LncRNA expression represents a potential prognostic marker for leukemia, and may enable risk stratification in leukemia patients. Feng et al. identified three lncRNAs (RP11-305O.6, AC092580.4, and RP11-222K16.2) related to the OS of AML patients, with further experiments suggesting that RP11-222K16.2 might affect the differentiation of natural killer cells to promote immune escape of AML 132. Tsai et al. recruited 275 newly diagnosed non-M3 AML patients and established a prognostic lncRNA score system comprising 5 lncRNAs, demonstrating lncRNA score as an independent prognostic factor for AML 133. Garzon et al. used a custom microarray platform to study lncRNA-expression profiles in elderly patients with normal cytogenetic (CN)-AML, with an emphasis on evaluating associations with conventional mutations and phenotypes. This led to the identification of dysregulated lncRNAs associated with select gene mutations and clinical outcomes 134. Additionally, they obtained a lncRNA score from 48 lncRNAs, with patients who had unfavorable lncRNA scores also displaying lower CR rates and shorter disease-free survival and OS. Because the clinical features, molecular abnormalities, and outcomes of older patients with CN-AML differ from those in younger adults, they also studied the prognostic value and biological significance of lncRNA expression in 377 adult patients (<60-years old) with CN-AML. Their results revealed no overlap between the 48 prognostic lncRNAs found in elderly CN-AML patients and the 24 transcripts reported in younger patients 135. This might be attributable to additional biological differences between the two cohorts, such as age-dependent differences in the frequency of mutations in recurrent prognostic genes. Mer et al. used RNA-seq analysis to detect lncRNA expression in 274 AML patients, finding that 33 lncRNAs were associated with OS^136^. A study identified 24 lncRNA signatures showing differential expression in CLL relative to normal B cell controls, with an independent risk model based on the expression of lnc-KIAA1755-4 and lnc-IRF2-3 capable of distinguishing between three different prognostic groups 137.

## Conclusions and Future Directions

LncRNAs are a large class of transcripts that play important roles in biological processes in malignant cells. The number of identified human lncRNAs has increased in the previous 15 years, with many of these also identified in leukemia. However, an understanding of the roles played by lncRNAs in leukemia occurrence and development remains insufficient. In this review, we briefly describe lncRNAs involved in leukemia progression and the underlying mechanisms.

LncRNA functions include regulation of cell differentiation, energy metabolism, malignant proliferation, and drug resistance. Future work should explore these functions more extensively, including subcellular localization to promote function prediction. Most lncRNAs localized to the nucleus modulate transcription and epigenetic modification, whereas those localized to the cytoplasm are likely to be involved in regulation at the post-transcriptional level. Further in-depth investigation of abnormally expressed lncRNAs in leukemia will enable elucidation of leukemia pathogenesis, and potentially provide feasible approaches for its treatment. Additionally, lncRNAs represent potential biomarkers for leukemia diagnosis and prognosis, with reports that abnormal expression of specific lncRNAs is related to leukemia-specific clinicopathological parameters. For example, HOTAIR and H19 correlate with poor OS in AML cases. Furthermore, model scores constructed based on differentially expressed lncRNAs obtained from sequencing or microarray data represent good prognostic predictors. Accordingly, expression of specific lncRNAs could represent a novel diagnostic biomarker and provide guidance for the prediction of clinical outcomes of leukemia; however, unified evaluation criteria based on a large sample are required. Prior to their use as leukemia biomarkers, large-scale prospective trials should be conducted in order to confirm their clinical usefulness and verify their accuracy and sensitivity as diagnostic and prognostic tools.

Although numerous lncRNAs have been identified, their regulatory functions remain largely unclear. Because lncRNAs exhibit low levels of expression and show poor species conservation relative to protein-coding genes, rapidly evolving RNA-seq technology can be used as a faster and more accurate detection system. Laboratory studies show that siRNA-targeting technology and CRISPR-Cas9 can effectively knockdown lncRNAs. The potential clinical utility of lncRNA-interference therapy should be explored in animal models and clinical trials in order to develop novel therapeutic strategies based on lncRNA targets in leukemia.

## Figures and Tables

**Table 1:** Aberrant LncRNA expression in different subsets of leukemia

Disease	LncRNA	Expression level in patients/cell lines	Mechanism	Clinical parameters and treatment responses	Ref.
AML	MEG3	Downregulated	Inhibits tumorigenesis in a p53-dependent and -independent manner	Abnormal methylation of MEG3 confers worse OS	23, 24
	H19	Upregulated	Possibly correlated with ID2 expression	Highest in M2 AML, correlated with sex, older age, higher WBC counts, intermediate karyotype, FLT3-ITD DNMT3A mutations, lower CR rate, and shorter OS	25.
	UCA1	Upregulated	Sponges for miR-126, miR-125a, miR-16; activates PI3K/AKT and JAK/STAT signaling	Elevated in patients carrying CEBPA mutations; elevated in ADR-resistant pediatric AML cases	26-28
	HOTAIR	Upregulated	Sponges for miR-193a and modulates c-KIT expression; regulates LSC self-renewal	Higher peripheral leukocyte and BM blast counts, lower platelet and hemoglobin counts, poor DFS and OS	29-33
	CRNDE	Upregulated	Promotes cell proliferation and cell cycle progression, inhibits apoptosis	Higher in M4 and M5 than in M1, M2, and M3 patients; negatively correlated with total survival time.	34
	PANDAR	Upregulated	Interacts with NF-YA and inhibits pro-apoptotic gene expression	Older age, higher BM blasts, poor karyotypes, lower CR rate, and shorter OS.	35
	PVT1	Upregulated	Sponge for miR-1204 and regulates MYC activation	Elevated in t (8;21) AML and APL. Corelated with high-risk clinical criteria; shorter LFS and OS	36-39
	CASC15	Downregulated	Regulates SOX4 expression	Elevated in t (8;21) AML, relatively better prognosis	40
	IRAIN	Downregulated	Interacts with the IGF1R promoter	Higher WBC counts, blast counts and shorter RFS, OS; refractory response to chemotherapy	41, 42
	RUNXOR	Upregulated	Interacts with the H3K27 methylase EZH2 and RUNX1	Elevated in t (8;21) AML	43
	CCAT1	Upregulated	Represses monocyte differentiation and promotes AML cell growth by sequestering miR-155	Significantly elevated in M4 and M5 subtypes	36, 44
	CCDC26	Upregulated	Regulates AML cell proliferation via c-KIT expression	Older age, anemia, poor/intermediate risk, partial/no remission, shorter OS	45, 46
	TUG1	Upregulated	Targets AURKA and induces AML cell proliferation; reduces miR-34a expression and contributes to ADR resistance	Higher WBC counts, FLT3-ITD mutation, monosomal karyotype, poor-risk stratification, and worse event-free survival and OS	47, 48
	MALAT	Upregulated	Influences proliferation, apoptosis and Ara-C sensitivity by upregulating miR-96	Markedly upregulated in M5 subtype, correlated with higher WBC and platelet counts, shorter OS	49, 50
	HOXA-AS2	Upregulated	Suppresses ATRA-induced apoptosis via TRAIL; increases ADR resistance via the miR-520c-3p /S100A4 pathway		51, 52
	MONC	Upregulated	Enhances proliferation of immature erythroid progenitor cells		53
	NEAT1	Downregulated	Impairs myeloid differentiation, regulates miR‐23a‐3p/SMC1A		54
ALL	BALR-2	Upregulated	Inhibits downstream glucocorticoid receptor genes FOS, JUN, and BIM	Shorter OS and poor response to prednisone	55
	BALR-6	Upregulated	Regulation of the transcriptome downstream of SP1	Highest expression in patients carrying MLL rearrangement	56
	CASC15	Downregulated	Regulates SOX4 expression	Elevated in pediatric B-ALL with t (12; 21); associated with relatively better survival	40
	GAS5	Downregulated	Sponge for miR-222; modulates B lymphocytic leukemia cell tumorigenesis and metastasis; essential for mTOR-related inhibition of T cell proliferation	Elevated on day 15, but decreased on day 33 after glucocorticoid therapy	57-59
	HOXA-AS2	Upregulated	Enhances glucocorticoid resistance, upregulates HOXA3 to activate EGFR/Ras/Raf/MEK/ERK signaling	Higher in pediatric prednisone-poor response ALL cases	60
	ZEB1-AS1	Upregulated	Promotes activation of IL-11/STAT3 signaling	Correlated with poor prognosis	61
	NEAT1	Upregulated	Related to dysregulation of miR-335-3p and indirectly regulates multidrug-resistance genes		62
	PVT1	Upregulated	Participates in cell cycle progression and proliferation regulation		63
	SNHG16	Upregulated	Host of miR-124-3p and promotes ALL cell proliferation		64
	NALT	Upregulated	Regulates NOTCH1 signaling		65
	T-ALL-R-LncR1	Upregulated	Inhibits formation of the Par-THAP1 complex and caspase-3 activation		66
	LUNAR1	Upregulated	Enhances IGF1R expression to sustain IGF1 signaling		67
	XLOC_001561	Downregulated	Involved in T cell differentiation and possible tumorigenesis		68
	ANRIL	Upregulated	Alters CDKN2A/B expression; targets EZH2, and activates the NF-κB pathway		69, 70
	Linc-PINT	Downregulated	Induces HMOX1 transcription and reduces ALL cell viability		71
	Lnc-INSR	Upregulated	Promotes immune suppression by enhancing Treg cell differentiation		72
	ARIEL	Upregulated	Activates ARID5B expression, thereby upregulating TAL1-induced transcriptional programs and MYC oncogene expression		73
	RP11-137H2.4	Upregulated	Involved in proliferation, apoptosis, cell migration, and glucocorticoid resistance		74
CML	HOTAIR	Upregulated	Contributes to IM resistance by activating the PI3K/AKT pathway	Upregulated in MRP1-high patients	75
	MEG3	Downregulated	Regulates miRNA-21, miRNA-147, and the JAK/STAT pathway	Lower in AP and BP than in CP patients. Lower in imatinib-resistant compared to imatinib-sensitive patients	76-78
	H19	Upregulated	Required for BCR-ABL-mediated tumorigenesis	A tendency toward higher WBC counts and BCR-ABL transcript	79, 80
	HAND2-AS1	Upregulated	Host of miR-1275 and promotes CML cell proliferation	Expression level in AP/BP stages was much lower than that in CP	81
	HULC	Upregulated	Sponge for miR-200a and modulates c-Myc and Bcl-2 levels	Positively correlated with clinical stages	82
	MALAT1	Upregulated	MALAT1/miR-328 axis promotes CML cell proliferation and imatinib resistance		83
	NEAT1	Upregulated	Essential mediator of apoptosis induced by imatinib		84
	SNHG5	Upregulated	Affects DR4 methylation; promotes IM resistance by attenuating miR-205-5p expression		85, 86
	UCA1	Upregulated	Sponge for miRNA-16 and contributes to IM resistance		87
	PLIN2	Upregulated	Promotes tumor growth via Wnt/β-catenin signaling		88
	FENDRR	Downregulated	HuR/FENDRR/miR-184 interaction contributes to MDR1 activity		89
	BGL3	Downregulated	Host of miR-17, miR-20a, miR-20b, miR-93, miR-106a, and miR-106b; regulates PTEN expression		90
CLL	DLEU1/2	Downregulated	Host of miR-12a and miR-16-1; regulates NF-κB signaling	Corelated with poor prognosis	91-94
	MALAT1	Upregulated	Involved in tumorigenic processes	No statistically significance difference between the prognosis categories	95
	MIAT	Upregulated	Initiates a regulatory loop with OCT4 in malignant mature B cells	Correlated with rapid death cases	96
	GATA6-AS1	Downregulated	Inhibits cell proliferation and enhances apoptosis in the caspase-9-dependent intrinsic apoptosis pathway	Methylation of GATA6-AS1 corelated with advanced Rai stage	97
	TRERNA1	Upregulated	Enhances protection against cytotoxicity mediated DNA damage	Associated with aggressive disease markers, shorter time to treatment, shorter PFS and OS	98
	lncRNA-p21	Downregulated	Activated by p53 and binds hnRNP-K to induce apoptosis		99, 100

## Abbreviations

ADR: doxorubicin
ALL: acute lymphoblastic leukemia
AMKL: acute megakaryoblastic leukemia
AML: acute myeloblastic leukemia
AMPK: Adenosine monophosphate-activated protein kinase
ANRIL: Antisense noncoding RNA in the INK4 locus
APL: acute promyelocytic leukemia
ARIEL: ARID5B-inducing enhancer associated long noncoding RNA
ATL: acute T cell leukemia
ATRA: all-trans retinoic acid
BALR: B-ALL associated long RNA
BGL3: β globin locus transcript 3
BP-ALL: B-cell precursor ALL
CASC15: cancer susceptibility candidate 15
CCAT1: colon cancer associated transcript-1
CDKN: cyclin-dependent kinase inhibitor
CEBPA: CCAAT enhancer binding protein α
CLL: chronic lymphocytic leukemia
CML: chronic lymphocytic leukemia
CN-AML: normal cytogenetic-AML
CRNDE: colorectal neoplasia differentially expressed
DLEU: deleted in leukemia
EZH2: enhancer of zeste homolog 2
FENDRR: FOXF1 adjacent non-coding developmental regulatory RNA
GAS5: growth arrest-specific 5
GATA6-AS1: homo sapiens GATA6 antisense RNA 1
H19: H19 imprinted maternally expressed transcript
HANDS-AS1: HAND2 antisense RNA 1
HK2: hexokinase 2
HSC: hematopoietic stem cell
HOTAIR: HOX antisense intergenic RNA
HOXA-AS2: HOXA cluster antisense RNA 2
HULC: highly upregulated in liver cancer RNA
LFS: leukemia-free survival
IGFR1: insulin-like growth factor receptor 1
IRAIN: IGF1R antisense imprinted non-protein coding RNA
JAK: Janus kinase
LincRNA-p21: long intergenic non-coding RNA p21
Linc-PINT: long intergenic non-protein coding RNA, p53 induced transcript
Lnc-INSR: lnc-insulin receptor precursor
LncRNA: long noncoding RNA
LSC: leukemia stem cells
LSD: leukemia stem cells
LUNAR1: leukemia-induced noncoding activator RNA-1
MALAT1: metastasis-associated lung adenocarcinoma transcript 1
MEG3: maternally expressed 3
MIAT: myocardial infarction associated transcript
mRNA: messenger RNA
miRNA: microRNA
MONC: megakaryocytic oncogenic non-coding RNA
ncRNA: non-coding RNA
NEAT1: nuclear paraspeckle assembly protein 1
NF-YA: nuclear paraspeckle assembly protein 1
PANDAR: promoter of CDKN1A antisense DNA damage activated RNA
PML-RARα: promyelocytic leukemia-retinoic acid receptor α
PRC2: Polycomb repressive complex 2
PVT1: plasmacytoma variant translocation 1
REST: RE1-silencing transcription factor
siRNA: small-interfering RNA
SIRT1: sirtuin-1
SMC1A: structural maintenance of chromosome 1α
SNHG: small nucleolar RNA host gene
SP1: Specificity Protein 1
STAT: signal transducer and activators of transcription
TRERNA1: translation regulatory long non-coding RNA 1
TUG1: taurine up-regulated 1
UCA1: urothelial carcinoma-associated 1
Xist: X-inactive-specific transcript
ZEB1-AS1: ZEB1 antisense RNA 1
